# Estimation of Notched Composite Plates Fatigue Life Using Residual Strength Model Calibrated by Step-Wise Tests

**DOI:** 10.3390/ma11112180

**Published:** 2018-11-03

**Authors:** Paweł Romanowicz, Aleksander Muc

**Affiliations:** Institute of Machine Design, The Cracow University of Technology, ul. Warszawska 24, 31-155 Cracow, Poland; olekmuc@mech.pk.edu.pl

**Keywords:** fatigue, life prediction, circular hole, elliptical hole, glass/epoxy composite plates

## Abstract

The proposed new technique of fatigue life prediction for notched composite plates is based on a residual strength model calibrated with the use of step-wise fatigue tests. It was proposed to calibrate the fatigue model with fatigue tests in which load conditions are in a step-wise fashion. The adopted fatigue model takes into account the most important loading parameters such as testing frequency, stress ratio, layer orientation and maximal fatigue stress. It was demonstrated that with the use of step-wise fatigue tests, it is possible to calibrate the fatigue model for a particular material and structure with the use of fewer samples. In the experimental tensile and fatigue tests TVR 380 M12/26%/R-glass/epoxy composite plates [+45°/−45°]_4_ with circular and elliptical cut-outs were used. The fatigue tests were performed under different loading conditions. The influence of testing frequency, stress ratio, maximal fatigue load and also geometry of the cut-out on damage growth rate and fatigue life were studied. The predicted fatigue life was in good agreement with the durability determined experimentally in all investigated samples.

## 1. Introduction

Multilayered fibre-reinforced composite materials are extensively used in various engineering applications including critical structural components. The most widely used for manufacturing of structures and machine components are graphite/epoxy and glass/epoxy composites. Due to their high strength/weight and high stiffness/density ratios, good chemical resistance and good fracture toughness they are used for components of automotive, aerospace structures, wind turbines blades, etc. Moreover, composite structures can be easily optimized to improve their mechanical properties (strength, stiffness, buckling resistance) with simultaneous reductions of cost and weight [1]. Composite structures are usually exposed to different loading conditions, such as static, fatigue or impact loadings. In the regime of cyclic loadings, durability and fatigue life prediction is an important issue. Generally, structures made of unidirectional composites are brittle and behave linearly under fatigue tensile loading. It may result in sudden failure of a structure subjected to cyclic load without any prior notification. Such effects are observed in the investigated glass/epoxy laminates with cut-outs subjected to cyclic tension [2]. The fatigue failure of composite structures can be described by a combination of different forms of damage such as fibre breakage, matrix cracking, delamination, ply failure or debonding [3,4,5]. All the above damage modes cause changes in the mechanical properties of lamina. Such mechanisms can be intensified by stress concentrations caused by factors such as manufacturing defects, cut-outs, notches and flaws. The phenomenon of stress concentration in thin plates with a cut-out and modelling of fatigue damage under unidirectional loading using different fatigue models was recently discussed in many papers [6,7,8,9,10,11,12]. Some experimental investigations of fatigue behaviour of notched composite plates under multiaxial loading condition were also performed [13,14,15,16,17,18].

The fatigue life and damage propagation in fibre-reinforced composite materials highly depends on a few parameters [19,20,21,22,23,24,25,26,27] among which the most important are orientation of fibres in respect to the loading direction (θ), frequency of fatigue loading (*f*), stress ratio (*R*), maximal applied stress (σ_max_) and temperature. The other parameters which affect static and fatigue behaviour are mechanical properties of matrix and fibre material, volume fractions, moisture content, porosity, etc. The influence of stress ratio (*R* = 0.1, *R* = 0.5 and *R* = −1) and stacking sequence for glass/polyester and glass/carbon/polyester laminates were investigated by De Smet and Bach [28]. They observed a linear relationship between stresses and stiffness degradation for laminates made of off-axis layers. Fatigue tests for specimens made of glass/epoxy composite under different cyclic loadings (*R* = 0.1–0.5) were done by Roundi et al. [29]. They observed that an increase of *R* in a tension-tension loading results in an increase of the fatigue life of composite structures [12,30,31,32]. However, an increase of *R* in a compression-compression case leads to the reduction of fatigue life [30,31]. Another parameter which has a significant effect on fatigue life is loading frequency *f*. Many researchers [20,30,33,34] observed that an increase of *f* decreases the damage propagation rate and in consequence increases the fatigue life.

The results of experimental tests are commonly represented using S–N curves. Generally, the slope of S–N curve of composite materials is very low or even quite flat in compression-compression tests. These can be characterised as good fatigue properties but such material response results in great sensitivity to the load level. Composite materials can also be characterised by poor fatigue resistance to compression loadings.

**Figure 1:**
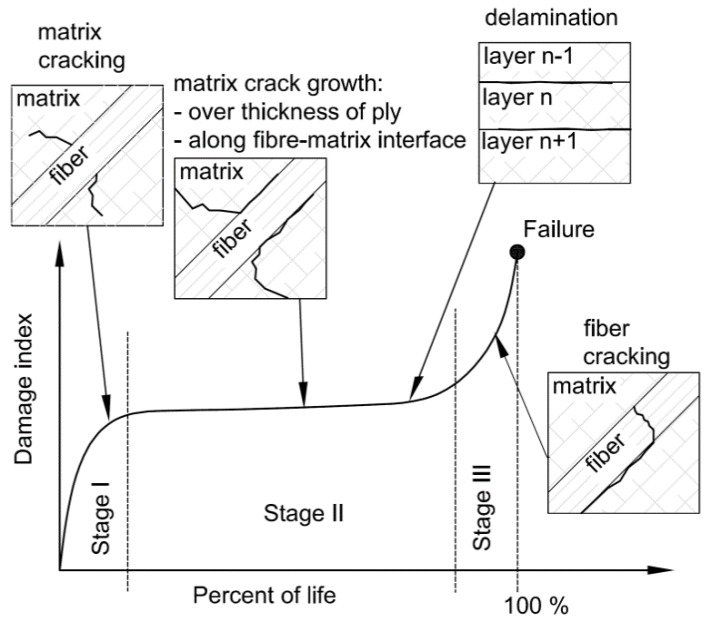
Damage mechanisms in unidirectional composite materials.

Generally, three different stages of the damage mechanism in the fibre-reinforced composites exposed to tension-compression cyclic loading can be distinguished (Figure 1) [35,36]. In the stage I diffuse damage of matrix is observed. It was revealed that damage develops rapidly to a great extent [37]. Initial cracks, which are formed in the matrix, slightly reduce the mechanical properties of a structure [38]. Such microcracks initiate at local micro-defects such as voids, resin-rich regions, misaligned fibres, etc. [39]. These cracks grow during fatigue life until they encounter a fibre. Further increase of cracks length (stage II) is typically observed over the thickness of the play and along the fibre-matrix interface. At this stage, with the largest and linear lifespan, the rate of damage growth and stiffness degradation is reduced. At the end of this stage, local delamination between layers may occur. Stage III is the shortest one which witnesses fibre cracking in the damaged zone. It results in rapid degradation of stiffness. The damage mechanisms for glass-fibre reinforced polymers are described in detail by Kennedy et al. [40]. The characteristic feature of damage curve for composite materials subjected to constant loading conditions is that stiffness reduction at stage II is approximately linear with respect to the number of cycles (Figure 1) [32,35,36].

Structural health monitoring [41], fatigue damage modelling and prediction of the fatigue life of structural components are significant engineering issues, mainly in aerospace and automotive applications. For isotropic materials, such as steel alloys, fatigue life can be estimated using the multiaxial high-cycle fatigue criteria. Generally, these models require only two classical material parameters—the alternate bending fatigue strength and the alternate torsion fatigue strength [42]. With the use of these two material parameters and the multiaxial high-cycle fatigue criterion, it is possible to estimate the fatigue life of an element made of an isotropic material under different loading conditions [42]. In the case of anisotropic and composite materials, the prediction of fatigue life is much more complicated than for isotropic materials. The fatigue models for fatigue life prognosis of fibre-reinforced polymers can be divided into four categories: The residual strength models, the criteria based on the damage accumulation mechanisms (DAM), the macroscopic strength fatigue criteria and the criteria based on residual stiffness [43]. Similar major categories of fatigue models are often proposed for multi-layered composite structures: The phenomenological criteria for the residual strength or stiffness, the progressive damage criteria and the fatigue life models based on the Goodman-type diagrams or S–N curves [44]. Generally, such models contain different parameters and sometimes require variable amplitude fatigue data of the investigated material [27,45]. Moreover, such criteria very often are limited to the specific loading conditions or materials. The detailed reviews of the fatigue life models can be found in References [44,46,47].

The fatigue models proposed in the literature very often require fitting of the failure curve to the experimental tests for an appropriate geometry and loading conditions. It means that the application of the mathematical model for the estimation of fatigue life requires costly and time-consuming experimental fatigue tests. However, there are cases in which the machine element is working under various cyclic loadings (stress ratio *R*, frequency *f*, maximal fatigue stress, etc.) and there is not enough time for designation of S–N curves for particular loading conditions. In such case, one of the alternatives may be the determination of fatigue limits using the step-wise fatigue testing [48,49,50,51,52,53]. This method, based on fatigue testing with increasing loading parameters during fatigue test, allows the determination of the fatigue limits with the use of a smaller number of specimens. An example of the step-wise technique application for damage characterization under static and fatigue tests of glass fibre-reinforced polymer composites can be found in References [51,52].

The aim of the present paper is to propose a method for quick determining of fatigue life of multilayered fibre-reinforced composite plates with notches for which experimental fatigue tests have not yet been performed. The proposed procedure is based on the observation of the fatigue behaviour of glass/epoxy composite materials under different fatigue loading conditions with the use of step-wise fatigue tests [48,49,50]. The application of dynamic step-wise testing with the use of infrared thermography for determination of fatigue strength and fatigue life was proposed by Colombo et al. [51]. They observed that this technique can be used for the estimation of fatigue behaviour of glass/epoxy laminates. A similar method is implemented for prediction of fatigue life with the use of the residual strength model. The largest difference is that during fatigue tests different loading parameters are changed. The advantage of the proposed technique is that it requires a smaller number of experimental tests for determining and fitting the failure curve of the fatigue model.

The paper consists of six sections. In Section 2 the fatigue life model based on the residual strength is discussed. The most important studies in which the model has been verified have been cited. The detailed information about the investigated glass/epoxy composite material is described in Section 3. The experimental tensile tests were performed for samples with different layers orientations—[0°]_8_, [90°]_8_ and [45°/−45°]_4_. The calibration of the fatigue life model is presented in Section 4. It was established using the tensile and step-wise fatigue tests for a plate with a circular cut-out. In Section 5 the experimental fatigue tests for plates with circular and elliptical cut-outs are discussed. The obtained results are compared with the fatigue life prediction. Conclusions are given in Section 6.

## 2. Fatigue Life Model

Fatigue damage in composite structures can be modelled adopting various approaches. The most popular fatigue life models for composite materials are based on the residual strength and the residual stiffness degradation [54] or damage mechanisms theories. These criteria are based on the phenomenon that the development of fatigue damage results in changes of the composite materials properties. Deterministic equation between the initial stiffness and the residual strength after *n*-cycles was proposed by Sendeckyj [43]. The residual stiffness fatigue models show the most significant advantages among the models mentioned above. They disclose greater degradation of material properties under fatigue load than the residual strength model and the change of elastic properties can be easily measured using non-destructive techniques [55,56]. The fatigue residual strength/stiffness models recently proposed in the literature [12,18,36,37,44,46,47,54,57,58,59,60,61,62] take into account different loading parameters. Some of the fatigue models are proposed for constant amplitude loadings or include only selected loading parameters. However, the structural components are generally subjected to variable amplitude loads with different frequencies and stress ratios. Consequently, the fatigue model for fatigue life prediction is selected on the basis of loading parameters included in the mathematical formulations. In the presented study it is assumed that the fatigue model should include the influence of the maximal magnitude of the applied fatigue stresses, stress ratio *R*, fibre orientation and loading frequency *f*. The above conditions are fulfilled, for instance, in the criteria proposed by Epaarachchi and Clausen [61] or Zhang et al. [54]. The most important practical difference between these two models is that the model proposed by Zhang et al. [54] requires more material parameters. 

The rate of strength degradation under fatigue loading can be described by the deterministic equation [63,64]:

(1) $$\frac{d\sigma }{dn}=-C_{1}n^{-m_{1}},$$

where σ is the residual strength degradation after *n* fatigue cycles and *C*_1_ and *m*_1_ are constants.

Integration and derivation of the Equation (1) was made by D’Amore et al. [63]. The model proposed by Epaarachchi and Clausen [61] is proposed for fibre-reinforced composite material under constant amplitude loading and is the extension of the formulation of the residual strength degradation model given by D’Amore et al. [63,64]. They assumed that *C*_1_ is the function of the different parameters such as stress ratio, the maximal stress, frequency, etc. The detailed considerations regarding the derivation of this model can be found in Reference [61]. The Epaarachchi and Clausen model [61] is defined as follows:

(2) $$\alpha \left(N_{f}^{\beta }-1\right)=\left(\frac{\sigma_{u}}{\sigma_{\max}}-1\right){\left(\frac{\sigma_{u}}{\sigma_{\max}}\right)}^{0.6-\psi \left|\sin\left(\theta \right)\right|}\frac{1}{{\left(1-\psi \right)}^{1.6-\psi \left|\sin\left(\theta \right)\right|}}f^{\beta }$$

with

(3) $$\psi =\left\{\begin{array}{c} R\;\mathrm{for}\;-\infty \;<\;R\;<\;1-\mathrm{tension}-\mathrm{tension}\;\mathrm{and}\;\mathrm{tension}-\mathrm{compression}\\ 1/R\;\mathrm{for}\;1<R<+\infty -\mathrm{compression}-\mathrm{compression} \end{array}\right\}$$

and

(4) $$R=\frac{\sigma_{\min}}{\sigma_{\max}}$$

where *N_f_* is number of cycles to failure, σ_u_ is the ultimate stress of the material in the loading direction, σ_min_ and σ_max_ are the minimal and maximal applied fatigue stress in the loading direction, respectively, θ is the smallest angle between the loading direction and fibre direction, *f* is loading frequency (Hz), *R* is stress ratio and α and β are material constants determined from experimental fatigue tests. Values 0.6 and 1.6 in (2) are postulated by Epaarachchi and Clausen [61] to take into account influence of fibre angle on fatigue life. The θ is introduced to take into account the effect of fatigue behaviour of matrix in the case of laminates with absence of fibres in the loading direction. For laminates with multiple layers, in which, one or more layers have fibres in the loading direction the angle θ = 0°. Different fatigue behaviours of such laminates with different layer orientation (i.e., [0°/90°/90°/0°] and [0°/30°/30°/0°]) is taken into account in the model (2) by including the ultimate tensile stress of the virgin material in the loading direction (σ_u_).

The model proposed by Epaarachchi and Clausen [61] was verified experimentally by Satapathy et al. [18] and Toumi et al. [65]. Good agreement of fatigue life prediction using this model for E-glass/epoxy and graphite/epoxy notched composite materials under tension-tension [18,61,65] fatigue load was observed. The model is also positively verified for notched graphite/epoxy composite samples under uniaxial and multiaxial loading conditions [18]. In the investigated examples [18] the maximal error between the experimental and predicted residual strength of notched samples does not exceed 15%.

The application of the Epaarachchi and Clausen (2) model enables the prediction of the fatigue life of multi-layered composite structures subjected to cyclic loading taking into account the essential loading parameters. The determination of the S–N curves for the particular parameters (*R*, *f*, σ_max_, etc.) seems to be the most time-consuming and expensive. Therefore, in the current study, a new method for determination of the failure curve has been used and verified. The proposed method, based on step-wise fatigue testing, allows faster determination of the impact of particular parameters on the damage growth rate with the use of a smaller number of samples. The proposed technique is described in detail in Section 4. The accuracy of the presented method was compared with accuracy of other fatigue models [54].

## 3. Material Characterization

The experimental tensile and fatigue tests were performed on samples made of TVR 380 M12/26%/R-glass glass/epoxy material. The samples were made from prepregs with fibre density of 2.56 g∙cm^−3^ and epoxy resin density of 1.24 g∙cm^−3^. Autoclaving method and a vacuum bagging technique with underpressure 0.08 MPa were applied. The autoclave operations were carried out under operating pressure of 0.4 MPa. The samples were cured at 135 °C for two hours. The heating and cooling gradients were 2 °C/min. The average volume fraction of fibres V_f_ = 66.71% and matrix V_o_ = 33.29% in the laminate were obtained.

**Figure 2:**
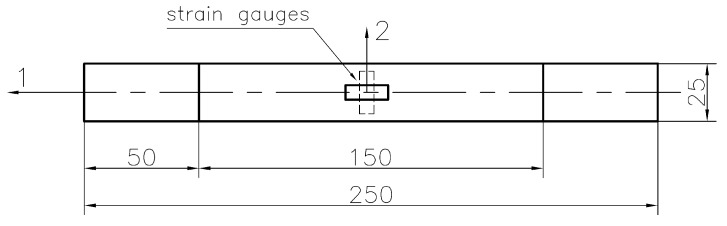
Geometry of specimen prepared for determination of mechanical properties and location of strain gauge sensors (dimensions in mm).

The mechanical properties of the investigated composite material were determined at room temperature. Twelve specimens (Figure 2) (four for particular orientation—θ = [0°]_8_, [90°]_8_ and [45°/−45°]_4_, where θ is the orientation of the fibres in relation to the tension direction) were tested. The samples consisted of 8 layers of average thickness of 2.12 mm. The strains were also controlled by strain-gauge measurements. The mechanical properties were calculated using strain gauges measurements. The critical strains and stress-strain characteristics were designated using strains measured by MTS Landmark testing machine (MTS Systems Corporation, Eden Prairie, MN, USA). The experimental tests were performed with a displacement rate of 3 mm/min. In all the tests good compatibility of strains measured by MTS Landmark servo-hydraulic testing machine and strain-gauges was obtained.

**Figure 3:**
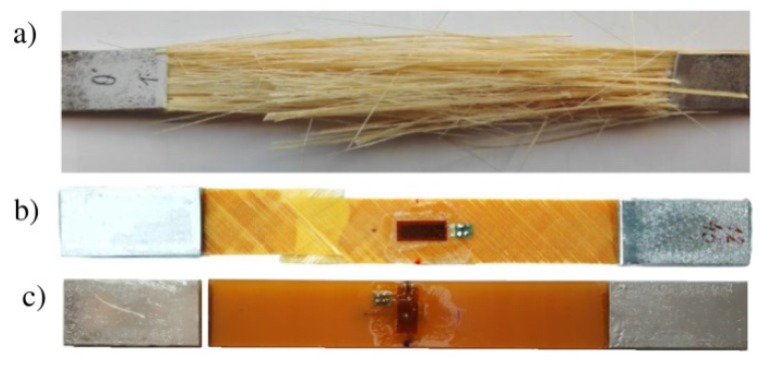
Failure forms of samples made of TVR 380 M12/26%/R-glass glass/epoxy material with layers orientation: (**a**) [0°]_8_, (**b**) [45°/−45°]_4_, (**c**) [90°]_8_.

Different failure mechanisms were observed in the investigated samples. The most visible included:

1. Explosive fracture [66] with fibre cracking (for θ = 0°—Figure 3a),
2. Plastic flaw and separation of the fibres from the matrix (for θ = ±45°—Figure 3b),
3. Matrix fracture (for θ = 90°—Figure 3c).

**Figure 4:**
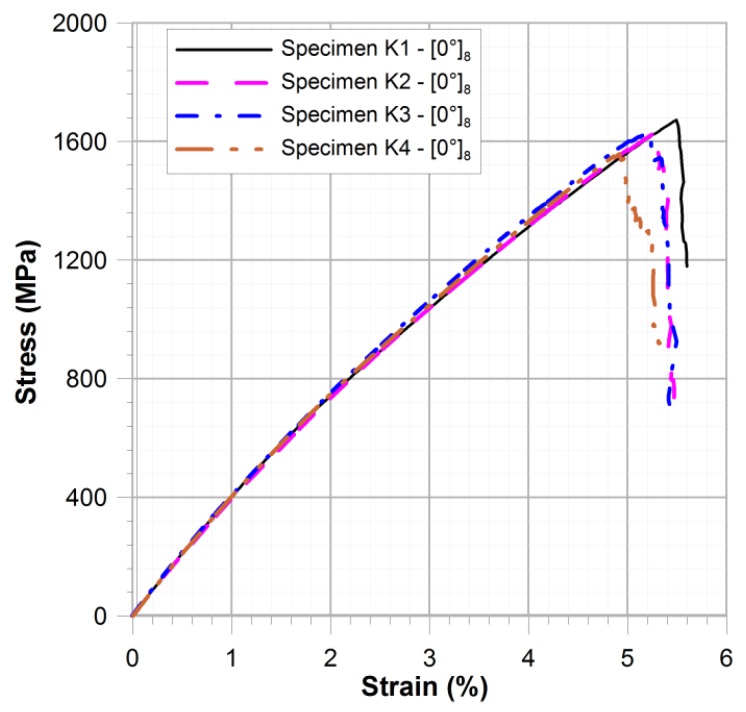
Comparison of stress-strain characteristics for specimens K1–K4 ([0°]_8_).

The failure of samples containing fibres parallel to the direction of load took the form of complete destruction of the matrix (Figure 3a). The cracking of the fibre was observed throughout the whole specimen. The determined tensile strength in the direction of fibres was in the range of 1546–1672 MPa (Figure 4—σ_max0°_ = {1546, 1559, 1627, 1672} MPa) and the average tensile strength was 1601 MPa.

**Figure 5:**
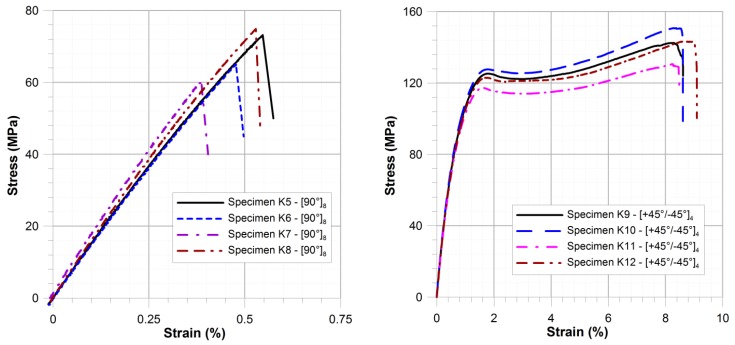
Comparison of stress–strain characteristics for specimens K5–K8 ([90°]_8_) and K9–K12 ([45°/−45°]_4_).

The failure mechanism of the specimens containing fibres perpendicular to the loading (θ = 90°) was significantly different (Figure 3c). The crack was located near to the overlay reinforcement and was caused by matrix fracture. The critical strain ε_1_^max^ was the order of magnitude less than for samples with θ = 0° and was 0.4–0.55%. The tensile strength of such samples was in the range of 60.4–74.9 MPa (Figure 5—σ_max90°_ = {60.4, 66.2, 73.3, 74.9} MPa) and average tensile strength was 68.7 MPa. In both above cases (θ = 0° and θ = 90°) the failure was rapid and the damage forms were repeatable.

The stress-strain curves for the samples with orientation [45°/−45°]_4_ are given in Figure 5. Three characteristic parts can be distinguished:—Tension, plastic flow and strengthening. In the first phase, the stress rapidly grew with even a slight increase of strain. Cracks in the matrix were formed from the beginning of the tensile test. The second phase started when tensile stress exceeded about 85% of the ultimate tensile strength. The slope of the curve was significantly changed. From this point, the strain began to increase with almost constant or slightly reduced stress. The delaminations, initiated at the free edge, were observed in the whole sample. In the third phase, the investigated material exhibited plastic strengthening. In the final phase of the test the layers were observed to separate and slide against each other. The process ended in the failure in the form presented in Figure 3b. The determined tensile strength was in the range of 130.5–150.8 MPa (Figure 5—σ_max45°/−45°_ = {130.5, 142.6, 143.3, 150.8} MPa) and the average tensile strength was 141.8 MPa. The obtained results of the above tests are given in Table 1.

## 4. Fatigue Model Calibration

The application of the fatigue models often requires the determination of S–N curves for corresponding loading conditions (*f*, *R*, fibre orientations, etc.) [18,45]. Such experimental tests allow the most accurate prediction of the fatigue response of the structure to be obtained. However, the determination of a larger number of S–N curves for various parameters at high resolutions of the investigated parameters becomes very expensive and time-consuming. It should also be noted that the fatigue life of multi-layered composite structures shows a large scatter [54]. The large interval of a number of cycles to failure under constant amplitude loading conditions is due to the statistical nature of fatigue of composites [63,64,67]. Therefore, calibration of the fatigue model using a limited number of experimental tests and probabilistic character of material degradation may result in differences between the experimental and predicted fatigue life. On the other hand, what is characteristic of multilayered composites is that an increase of strain ε = f(*n*) and damage *D* = f(*n*) during the cyclic load at stage II of material degradation (Figure 1) are approximately linear [36,57,68,69]. The nonlinear behaviour, which occurs at the initial stage I and the final part (stage III) is generally caused by matrix and fibres cracking, respectively (Figure 1). However, both nonlinear stages are relatively short in comparison with stage II. Moreover, stage III generally ends in abrupt failure of the specimen and the scale of nonlinearity depends on the number of damaged fibres. In the present study, material degradation is presented by means of a damage model defined using the Secant Young’s Modulus *F*(*n*). The model was proposed by Yang et al. [70] and given below

(5) $$D_{F}=1-\frac{F(n)}{F(n=0)}\;\mathrm{and}\;F(n)=\frac{\sigma_{\max}}{\varepsilon_{1}(n)}$$

where σ_max_ is the maximal fatigue stress and ε_1_(*n*) is the actual measured value of strains for the *n*-th cycle.

Damage parameter *D_F_* is a scalar and generally is defined as *D_F_* = 1 − φ, where φ is the magnitude of material degradation. For a material with no damages *D_F_* = 0, while *D_F_* = 1 corresponds to a state of complete failure. The development of stiffness degradation models is discussed in detail by Paepegem [36].

In view of the above, in order to reduce the number of samples and to reduce the time required for the determination of failure curve the following step-wise technique was implemented. The impact of particular parameters were studied employing the step-wise fatigue tests. For each loading condition, the strain growth rate was determined. The number of cycles to failure was expected by assuming the linear strain growth rate and failure of all cases at the level of the critical strain ε_1_^max^. The critical strain was assumed as that at fibre cracking occurrence (end of stage II). The initial strains were deduced from the tensile test. It should be emphasised that the proposed technique can be implemented only for materials in which the increase of strain under cyclic load under constant loading condition is linear and the failure strain must be independent of the loading condition.

The most significant advantage of this method is that it allows fitting the fatigue model for a specific material, geometry and loading conditions in much shorter time. However, such estimation may include a calculation error associated with extrapolation of ε = f(*n*) curve. On the other hand, in the constant amplitude fatigue test a considerable scatter of fatigue life is observed [54,60,63,64,67]. Consequently, performing a few constant amplitude fatigue tests does not necessarily guarantee a precise determination of the fatigue limit. For practical application, in which a few parameters of loading condition should be included in fatigue analysis, a large number of fatigue tests may be required. In such case, the proposed method can give significant benefits (smaller number of samples, shorter time of fatigue tests, etc.), with slightly lower accuracy of fatigue life predictions.

Fatigue life can be calculated after fitting the failure curve of the fatigue model to the investigated material, geometry and loading condition. The number of cycles to failure can be predicted using the residual strength (2) proposed by Epaarachchi and Clausen [61]. The formulation of the model is given as:

(6) $$N_{f}={\left[1+\frac{1}{\alpha }\left(\frac{\sigma_{u}}{\sigma_{\max}}-1\right){\left(\frac{\sigma_{u}}{\sigma_{\max}}\right)}^{0.6-\psi \left|\sin\left(\theta \right)\right|}\frac{1}{{\left(1-\psi \right)}^{1.6-\psi \left|\sin\left(\theta \right)\right|}}f^{\beta }\right]}^{\frac{1}{\beta }}$$

**Figure 6:**
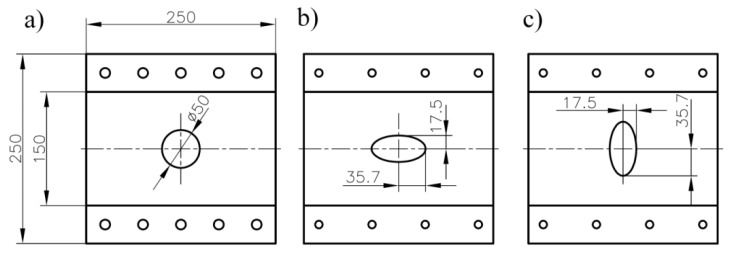
Geometry of the investigated composite plates with circular (**a**), and elliptical (**b**,**c**) holes at centre of plate (dimensions in mm).

The static tensile and fatigue tests were performed in order to fit the fatigue life model (6) to the investigated structures. The static tensile tests were conducted for composite plates with cut-outs located at the centre of plates. Three different shapes of holes were investigated—circular hole, and two elliptical ones of different orientations (Figure 6). The specimens consisted of eight layers in configuration [45°/−45°]_4_. The dimensions of the plate were 250 mm × 250 mm, and the average thickness of the plate was 2.12 mm. The samples were mounted in the load cells by means of additional instrumentation (grips for rectangular plate) and conducted with the use of the digital image correlation and the infrared passive thermography camera. More details about these fatigue tests can be found in Refs [2,55,71,72]. The measuring length was 150 mm.

**Figure 7:**
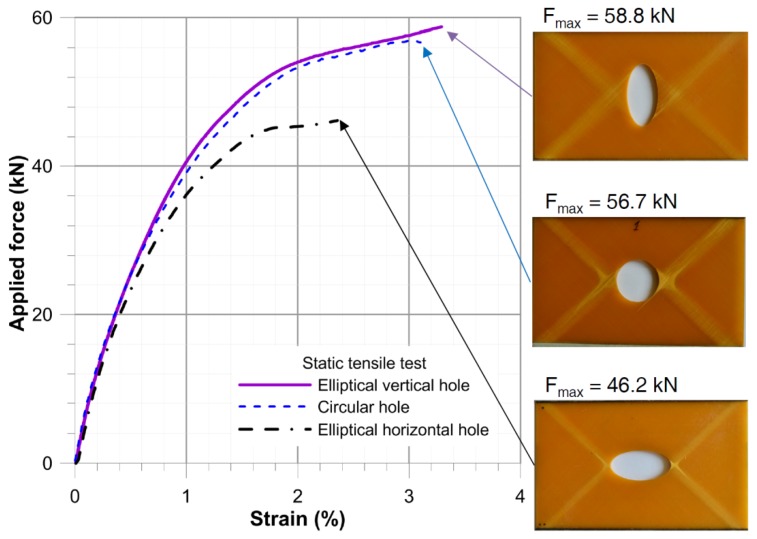
Static tensile tests for plates with circular and elliptical holes.

The failure forms for the static tensile tests (tensile rate *v* = 0.5 mm/min) for a composite plate with a hole is illustrated in Figure 7. In all the investigated cases, the first damage involved matrix cracking and occurred near the hole. Further loading resulted in the propagation of such cracks in the direction towards the corners of the plate, creating a distinctive sign “X”. At the point of intersection of crack bands further degradation of the structure followed. Simultaneously, delaminations at the edges of holes were initiated.

**Figure 8:**
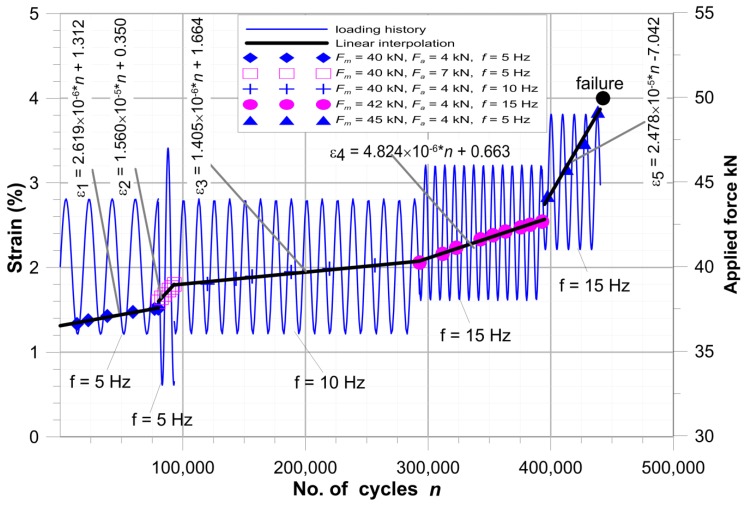
Step-wise fatigue test for a plate with circular hole (description in text).

The experimental step-wise fatigue tests under different loading condition were performed in order to fit the residual fatigue strength model (6) to the investigated structure and material. The amplitude of loading (4–7 kN), the mean value of tensile load (40–45 kN), stress ratio *R* and frequency *f* (5–15 Hz) were changed during the fatigue tests. All fatigue tests were monitored for effects from heating and results obtained from the infrared passive thermography are discussed in [71]. The detailed information about the performed step-wise fatigue tests are given in the Table 2. The effect of these parameters on the material degradation rate is illustrated by the ε–*n* diagram (Figure 8). The numbers of cycles *n*, for which the experimentally measured strains ε are given, were selected randomly. On the basis of the observations concerning multilayered composite materials degradation [36,40,57,68,69] and assumptions described in the chapter 4, each stage of the fatigue tests was described by means of linear interpolation in the form ε = *a*·*n* + *b*. It should be noted that only the first nonlinear part of the material degradation in test No.1 was disregarded in the analysis (see Figure 1). In all the cases the linear interpolation gives a good fitting to the experimental results. The particular equations in respect to the number of cycles *n* are given in the diagram. Fibre cracking occurs at the strain ε_1_^max^ ≅ 3.94%, which is assumed as the critical fatigue strain ε_1_^max^. From this point maximal force was observed to decrease and then rapid failure occurred after 40 cycles.

The slope of the ε–*n* curve, which was defined as tg(*a*) in the Table 2, is the most important parameter in the analysis and was used to evaluate the expected number of cycles to failure $N_{f}^{\mathrm{prog}}$. Such predictions were made with the following assumptions:

1. The fatigue failure occurs at the critical strain ε_1_^max^ = 3.94%,
2. The increase of strain under cyclic load with constant loading condition is linear,
3. Fatigue life prognosis was made using the strain after the static tensile test (Figure 7—as the initial strain under the maximal fatigue stress) and using the slope of the strain growth line under cyclic load tg(*a*) (Figure 8, Table 2—for determination of the duration of material degradation until the critical strain is achieved ε_1_^max^).

The expected fatigue lives for performed step-wise fatigue tests using the proposed technique are given in Table 2. It should be noted that fatigue of composites is a stochastic phenomenon and depends on the sequence of different damage accumulation mechanisms occurring at different length scales and the statistical distribution of defects [62]. The fatigue life of composite structures can vary even some two orders of magnitude [73]. This can lead to the large scatter of experimental number of cycles to failure and requires a statistical approach. Some procedures of statistical approach for strength degradation models are proposed by D’Amore et al. [64,67,73]. The number of cycles to failure can be also statistically evaluated using the fuzzy set approach [74,75].

The main aim of the paper is to present the application of step-wise fatigue tests for calibration of the fatigue model. The expected numbers of cycles to failure $N_{f}^{\mathrm{prog}}$, which are given in the Table 2, are evaluated using fatigue tests subjected to different loading conditions. In contrast to the statistical approach reported in papers [62,64,67,73,74,75], the expected fatigue life $N_{f}^{\mathrm{prog}}$ evaluated in the paper using single step-wise fatigue tests have a deterministic nature. It is obvious that for practical application, it is necessary to take into account statistical nature of fatigue phenomenon, loading history, environmental conditions, etc. This can be only achieved by performing fatigue tests for more samples. However, such an approach with single step-wise fatigue tests can be used i.e., for fatigue tests planning or preliminary fatigue tests.

The material constants for investigated plates, i.e., α = 0.123351 and β = 0.244873 were fitted to the results of fatigue tests under various loading conditions (Table 2) using the least square method. The ultimate stress of the material in the loading direction σ_u_ = 142 MPa was determined from tensile tests (Figure 5, Table 1). The obtained fatigue life prognosis for plate No.1 (given in Table 2) and calculated fatigue life using the fatigue life model (6) with the material constants α = 0.123351 and β = 0.244873 are compared in the Table 3. The relative difference was calculated as follows [54]:

(7) $$\delta_{1}={\left(\frac{\log N_{f}^{\mathrm{pred}}-\log N_{f}^{\mathrm{prog}}}{\log N_{f}^{\mathrm{prog}}}\right)}^{2}$$

The power 2 of the relative difference in (7) reduces the real differences for *δ*_1_ < 1.

It can be observed that the adopted fatigue life model (6) allows a sufficiently accurate adjustment of the failure curve for the considered loading conditions to be obtained.

**Figure 9:**
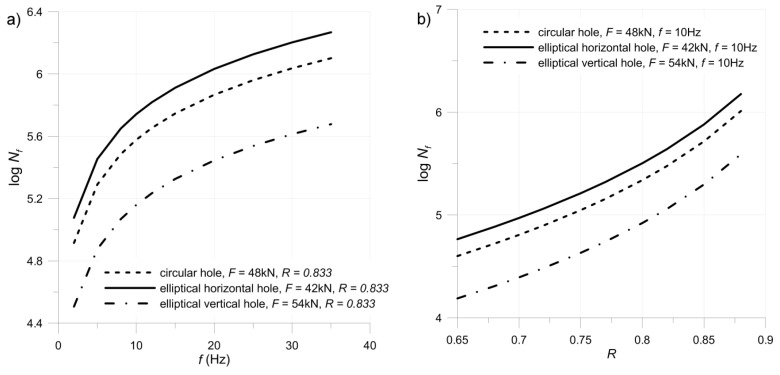
Influence of frequency (**a**) and stress ratio (**b**) on fatigue life prediction in the fatigue model (4).

The advantage of the residual strength model is that it takes into account the effect of different variables such as stress ratio, frequency, the orientation of layers and fatigue loading (Figure 9). The influence of testing frequency on damage growth rate is illustrated by the plate with circular hole (plate No. 1—Table 2). An increase of testing frequency (compare tests No. 1 and 3) results in a decrease of damage growth rate (decrease of tg(*a*)) and an increase of fatigue life. It can be observed that the fatigue model (6) can be fitted to predict this trend (Table 3). A more detailed study of the effect of the frequency of applied loading on damage growth rate is given in Reference [55]. The influence of the maximal applied load and stress ratio was also included in the fatigue model (details in Table 2).

## 5. Fatigue Tests

**Figure 10:**
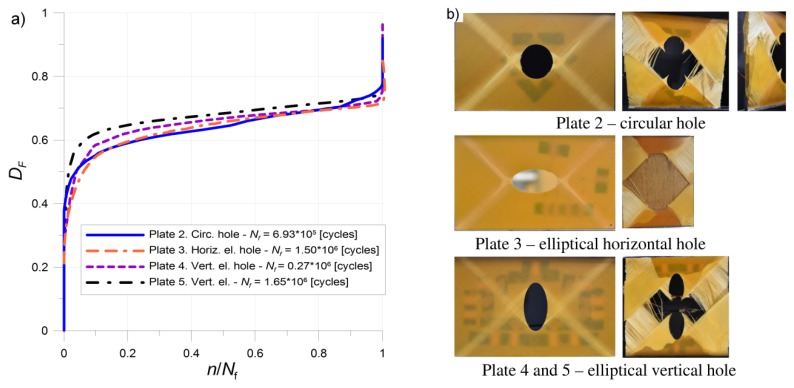
Damage growth (**a**) and failure forms (**b**) of investigated plates with various cut-outs.

Special attention is focused on the experimental fatigue tests of composite plates with circular and elliptical holes (Figure 6). The fatigue tests were performed for five plates with different cut-outs (Figure 6 and Figure 10). The loading condition and results of the fatigue tests are given in Table 4. The specimens were examined under a constant-amplitude loading condition. The second plate (No. 2) with a circular hole was loaded by a fatigue tensile force of the mean value of *F_m_* = 44 kN and amplitude *F_a_* = 4 kN. The failure form presented in Figure 10 occurred after *N_f_* = 692,565 cycles.

The plate with an elliptical horizontal hole (No. 3) was loaded by mean value *F_m_* = 38 kN and amplitude *F_a_* = 4 kN. The failure occurred after *N_f_* = 1,502,341 cycles. The plates with an elliptical vertical hole were loaded by mean value *F_m_* = 50 kN and amplitude *F_a_* = 5 kN (No. 4) and *F_a_* = 4 kN (No. 5). The slight change of the maximal load results, in this case, in a significant increase of fatigue life from *N_f_* (No. 4) = 270,061 cycles to *N_f_* (No. 5) = 1,651,871 cycles.

Degradation of the tested samples during the cyclic loading is presented using the damage parameter *D_F_* proposed by Yang et al. (5).

For all the tested plates with different cut-outs, the ε–*n* curve was almost linear in stage II of damage growth (Figure 10). It was also observed that stage III began when the damage level *D_F_* reached the value of 0.72–0.75. The final failures in all plates involved fibre fracture and were rapid.

Fatigue lives for the investigated plates with circular and elliptical cut-outs were predicted and given in Table 4 using the residual strength model (6) with the material parameters α = 0.123351 and β = 0.244873 established in Section 4 from the step-wise fatigue tests. The relative error *δ* (8) was calculated in reference to the logarithmic fatigue life of the experiment $\log N_{f}^{\exp}$ and the predicted logarithmic fatigue life $\log N_{f}^{\mathrm{pred}}$ using the formula proposed by Zhang et al. [54]:

(8) $$\delta ={\left(\frac{\log N_{f}^{\mathrm{pred}}-\log N_{f}^{\exp}}{\log N_{f}^{\exp}}\right)}^{2}$$

The fatigue life predicted using the proposed methodology was in good agreement with the results of the experimental tests. The maximal relative error was *δ* = 5.18 × 10^−3^. Obtained accuracy is comparable with more complicated models proposed in the literature and fitted by a larger number of test samples [54]. It should be noted that in the present paper, the fatigue model was adjusted using the step-wise fatigue experimental tests on only one sample. Such tests were performed with different stress ratios, the maximal applied stresses and frequencies for the plate with a circular hole. The determination of the damage growth rate under different loading conditions (five different tests) allows quite accurate fitting of the fatigue model also for structures with different shapes of cut-outs. The investigated plates (No. 2–5 in Table 4) have the same size and ply orientations. However, they differ in the notch shape. The influence of notch size and shape is included in σ_max_ in model (6). A limitation of this method is that it can be used only for materials with linear ε–*n* curve and failure strain must be independent of the loading condition.

## 6. Conclusions

A new technique of calibration of the fatigue model for notched composites with a fatigue tests in which load conditions are in a step-wise fashion is proposed in the paper. It was demonstrated that with the use of step-wise fatigue tests, it is possible to calibrate the fatigue model for particular structures subjected to the various loading condition with the use of fewer samples. The proposed technique for fatigue life prognosis with the use of the residual strength model, fitted to the investigated structure by means of step-wise fatigue tests, allows the prediction of the number of cycles to failure and takes into account different loading parameters, such as testing frequency, stress ratio, layer orientation and maximal fatigue stress. It was demonstrated that with the use of step-wise fatigue tests the fatigue model can be quickly and accurately fitted for various loading conditions simultaneously.

The experimental static and fatigue tests were performed for samples and plates made of glass/epoxy composite. The static tensile strengths for different orientation of fibres were determined. The influence of stress ratio, loading frequency and maximal load on damage growth rate was studied with the use of step-wise fatigue tests. The fatigue model was successfully calibrated using a single fatigue test in which load conditions occur in a step-wise fashion. The constant-amplitude fatigue tests were performed for composite plates with different shapes of cut-outs. In all the investigated samples the linear ε–*n* behaviors for stage II of damage growth were observed. Moreover, in all the investigated cases the failure occurred at a similar damage level of *D_F_*. Finally, the proposed technique of fatigue life predictions was applied to fatigue tensile tests of multilayered composite plates with circular and elliptical cut-outs. The predicted fatigue lives were in good agreement with the fatigue strengths determined experimentally for all tested samples.

Summarising, the application of step-wise fatigue tests enables fitting of the failure curve in a shorter time with the use of a smaller number of samples. This fact is fairly important in structures under potentially different loading conditions when quick estimation of the fatigue life of the structure is necessary. The proposed technique can also be used for fatigue tests planning.

## Figures and Tables

**Table 1:** Mechanical properties of TVR 380 M12/26%/R-glass glass/epoxy composite material.

E_1_ [GPa]	E_2_ [GPa]	ν_12_	Tensile Strength [MPa]			ε_1_^max^ [%]		
			[0°]_8_	[45°/−45°]_4_	[90°]_8_	[0°]_8_	[45°/−45°]_4_	[90°]_8_
46.4	14.9	0.27	1601	141.8	68.7	5.59	8.41	0.5

**Table 2:** Fatigue tests parameters and expected fatigue lives for performed step-wise fatigue tests for plate No. 1 with circular hole.

No.	Loading Parameters	Number of Executed Cycles	Increase of Strains in (%) Per 50 k Cycles	Tg(*a*)-Slope of Strain Growth Line	Expected $N_{f}^{\mathrm{prog}}$
1	*F_m_* = 40 kN, *F_a_* = 4 kN, *R* = 0.818, *f* = 5 Hz,	*N* = 0–80,000	0.131	2.619 × 10^−6^	1.045 × 10^6^
2	*F_m_* = 40 kN, *F_a_* = 7 kN, *R* = 0.702, *f* = 5 Hz,	*N* = 80,000–93,000	0.78	1.560 × 10^−5^	1.635 × 10^5^
3	*F_m_* = 40 kN, *F_a_* = 4 kN, *R* = 0.818, *f* = 10 Hz,	*N* = 93,000–293,000	0.07	1.405 × 10^−6^	1.95 × 10^6^
4	*F_m_* = 42 kN, *F_a_* = 4 kN, *R* = 0.826, *f* = 15 Hz,	*N* = 293,000–393,000	0.241	4.824 × 10^−6^	5.432 × 10^5^
5	*F_m_* = 45 kN, *F_a_* = 4 kN, *R* = 0.837, *f* = 15 Hz	*N* = 393,000–440,460	1.239	2.478 × 10^−5^	0.973 × 10^5^

**Table 3:** Comparison between expected and calculated fatigue lives for plate No. 1 with circular cut-out.

No.	Expected $\log N_{f}^{\mathrm{prog}}$ (Details in Table 2)	Calculated Using Model (6)—$\log N_{f}^{\mathrm{prog}}$, α = 0.123351, β = 0.244873	Difference *δ*_1_ (7)
1	6.02	5.81	1.2 × 10^−3^
2	5.21	4.71	9.5 × 10^−3^
3	6.29	6.10	9.2 × 10^−4^
4	5.73	6.02	2.5 × 10^−3^
5	4.99	5.59	14.8 × 10^−3^

**Table 4:** Fatigue tests parameters and comparison of determined and predicted fatigue life.

No.	Hole	Loading Parameters	Log (*N_f_*) Experiment	Log (*N_f_*) Predicted	Error *δ* (8)
2	Circular	*F_m_* = 44 kN, *F_a_* = 4 kN, *f* = 15 Hz	5.84	5.75	2.54 × 10^−4^
3	Elliptical horizontal	*F_m_* = 38 kN, *F_a_* = 4 kN, *f* = 30 Hz,	6.18	6.04	4.86 × 10^−4^
4	Elliptical vertical	*F_m_* = 50 kN, *F_a_* = 5 kN, *f* = 30 Hz	5.43	5.30	5.64 × 10^−4^
5	Elliptical vertical	*F_m_* = 50 kN, *F_a_* = 4 kN, *f* = 30 Hz,	6.21	5.77	5.18 × 10^−3^

## Nomenclature

tg(*a*)	slope of strain growth line
*D_F_*	damage parameter
*E_i_*	Young’s modulus in the *i* direction
*f*	loading frequency
*F_m_*, *F_a_*	mean value and amplitude of loading, respectively
*R*	stress ratio
*n*	number of loading cycles
*N_f_*	number of cycles to failure
$N_{f}^{\mathrm{pred}}$	predicted number of cycles to failure
$N_{f}^{\mathrm{prog}}$	expected number of cycles to failure
α and β	material constants
*δ*	relative error
*δ*_1_	relative difference
ε_1_^max^	critical strain in loading direction
σ_min_ and σ_max_	minimal and maximal applied fatigue stress in loading direction, respectively
σ_u_	ultimate stress of material in loading direction
ν_12_	Poisson’s ratio
θ	smallest angle of fibres between the loading direction and fibre direction

## Appendix A

Procedure for the step-wise characterization under fatigue loading:

1. Determination of the ultimate stress of material in loading direction σ_u_ for investigated laminate,
2. Determination of *F*–ε curves (static tension) for investigated notched composite structure, *F*-is applied force,
3. Determination of ranges of the loading parameters (the stress ratio *R*, frequency *f*, the maximal tensile load-σ_max_),
4. Step-wise fatigue tests,
5. Determination of the critical strain ε_1_^max^ for investigated structure,
6. Calculation of the slopes of strain growth lines tg(*a*),
7. Estimations of the $N_{f}^{\mathrm{prog}}$ using the strain after the static tensile test, tg(*a*) and ε_1_^max^,
8. Determination of the material constants α and β for the obtained data.
